# Proxy rated quality of life of care home residents with dementia: a systematic review

**DOI:** 10.1017/S1041610216002167

**Published:** 2017-01-16

**Authors:** Sarah Robertson, Claudia Cooper, Juanita Hoe, Olivia Hamilton, Aisling Stringer, Gill Livingston

**Affiliations:** Division of Psychiatry, University College London (UCL), 6^th^ Floor Maple House, W1T 7NF, London, UK

**Keywords:** dementia, quality of life, carers, nursing homes

## Abstract

**Background:**

Quality of life (QoL) is an important outcome for people with dementia living in care homes but usually needs to be rated by a proxy. We do not know if relative or paid carer proxy reports differ. We conducted the first systematic review and meta-analysis of data investigating whether and how these proxy reports of QoL differ.

**Methods:**

We searched four databases: Medline, Embase, PsychInfo, and CINAHL in October 2015 with the terms: *dementia, QoL*, *proxy*, and *care home*. Included studies either compared proxy QoL ratings or investigated the factors associated with them. We meta-analyzed data comparing staff and family proxy rated QoL.

**Results:**

We included 17/105 papers identified. We found no difference between global proxy ratings of QoL (*n* = 1,290; pooled effect size 0.06 (95% CI = −0.08 to 0.19)). Studies investigating factors associated with ratings (*n* = 3,537) found family and staff ratings correlated with the resident's physical and mental health. Staff who were more distressed rated resident QoL lower. Relatives rated it lower when the resident had lived in the care home for longer, when they observed more restraint, or contributed more to fees.

**Conclusions:**

Relatives and staff proxy QoL ratings share a clear relationship to resident health and overall ratings were similar. Rater-specific factors were, however, also associated with scores. Understanding why different raters consider the QoL of the same person differently is an important consideration when evaluating the meaning of proxy rated QoL. Proxy raters’ backgrounds may affect their rating of QoL.

## Introduction

The number of people living with dementia worldwide is estimated at 47.5 million and is projected to increase to 75.6 million by 2030 (World Health Organization and Alzheimer's Disease International, 2012). The World Health Organisation (1995) defines quality of life (QoL) as “an individual's perception of their position in life in the context of the culture and value systems in which they live and in relation to their goals, standards and concerns.” There is no cure for the dementias, so enabling people to live well with dementia is critical. Accurate measurements of QoL are a way of assessing whether this is happening and provide a means to evaluate the success of interventions designed to improve healthcare and help people live well with dementia. QoL is also a vital outcome for dementia research because older people often have multiple morbidities and, as a consequence, measuring individual targeted symptoms may mask a deterioration in other facets of health and not reflect the overall QoL of someone with dementia. Furthermore, in dementia, there may be no simple association between health-related QoL and an easily measurable clinical variable (Banerjee *et al*., 2009). It is, therefore, important to include QoL as an independent outcome for people with dementia and to consider our assessment of it.

QoL measurement is subjective and is ideally reported by the individual concerned. However, dementia impacts an individual's ability to understand abstract concepts, remember their feelings over the last hours, days or weeks and articulate their answers. Consequently, QoL data often needs to be obtained via a proxy: A person that knows the person with dementia well and is able to provide information. This information is considered valid based on their knowledge of the person, and current observations, about how the person is likely to be feeling in and experiencing their current situation. However, in community settings, research has found consistent differences between self-reported and proxy-reported QoL in this context, with self-reported QoL rated higher than proxy-reported QoL (Coucill *et al*., 2001; Novella *et al*., 2001; Selai *et al*., 2001; Logsdon *et al*., 2002; Ready *et al*., 2004). This suggests that proxies differ systematically from the person with dementia in how they evaluate their life quality.

In response to public concern about the QoL of people with dementia living in care homes, there have been substantial recent investments into research to improve it (NIHR, 2012; NIHR, 2015). In care home studies, proxy-reported QoL becomes more important as residents are more likely to have severe dementia than in the community (Beerens *et al*., 2014) and so be less likely to be able to self-report their life quality (Hoe *et al*., 2006). For the findings from this research and subsequent policy and interventions to be meaningful, it is essential that QoL measurements of residents with dementia are valid. It may be, however, that the evaluated success of an intervention in improving QoL depends on the perspective gathered (Goyder, *et al*., 2012) and that family relatives and care staff perceive intervention effects differently (Clare *et al*., 2013).

To inform the debate about how the QoL of care home residents with dementia can be most validly captured in research studies, we have systematically reviewed and meta-analyzed the evidence for how personal and professional proxies rate the overall QoL of care home residents with dementia, for the first time. We explore whether there are systematic differences in how relatives and paid staff of care home residents with dementia proxy rate QoL, by comparing the mean total score between groups, exploring the correlation between individual ratings and comparing the factors associated with QoL ratings between these groups.

## Methods

### Search strategy

The review was registered on the International Prospective Register of Systematic Reviews (PROSPERO) on the 18th February 2015. Searches were conducted in October 2015 in Medline, Embase, PsychInfo, and CINAHL databases; using the search terms: *dementia* AND *QoL* AND *proxy* AND *care home.* The variants on terms used were as follows: for the disease ((dementia$ OR alzheimer$); the participant perspective ((*proxy* OR *observer*$ or *informant*$ OR *carer*$ OR *caregiver*$ OR *care* OR *staff* OR *professional*) adjacent to (*rater*$ OR *rated* OR *rating*$ OR *report*$ OR *perspective*$)); the place of residence (((*residential* adjacent to (*care*$ OR *service*$ OR *facilit*$ OR *home*$)) OR (*care* adjacent to (*home*$ OR *service*$ OR *facilit*$ OR *home*$)) OR (*nursing* adjacent to (*care*$ OR *service*$ OR *facilit*$ OR *home*$)) OR *institution*$ OR “*group dwelling*$” OR “*long term care*”); and the outcome of interest (((*quality* adjacent to *life*) OR *well-being*) within seven words of (*measure*$ OR *scale*$ OR *survey*$ OR *questionnaire*$ OR *outcome*$)). We hand-searched the references of all included papers and contacted authors of included papers to ask about other related literature. Where information from papers was missing, we contacted the authors to ask for the information.

### Inclusion criteria

We included studies in any language reporting quantitative ratings of QoL of people with dementia living in care homes and either (1) comparing two different proxy perspectives for the same individual or (2) describing the factors associated with proxy rated QoL.

### Data extraction and validity rating

SR extracted data and SR, OH, and AS independently rated quality using operationalized checklists for quantitative papers previously developed by our group (Mukadam *et al*., 2011) from standardized assessment tools (Boyle, 1998) to assess quality. This included the following questions:

1. Was the population defined by a clear inclusion and exclusion criteria?
2. Were the data collection methods standardized?
3. Were the measures used for QoL valid, and reliable, and used in an appropriate way?
4. Was there sufficient power to conduct the analysis, judged by a sample size of greater than 30 where a power analysis had not been conducted?

We then met to discuss any discrepancies. We prioritized higher quality papers, defined as those meeting all the above criteria.

### Analysis

We used Stats direct version 3 to Meta-analyze data from studies that reported family carer and staff proxy QoL scores. We used the means and standard deviations of scores to calculate the pooled effect size and confidence intervals using the DerSimonian Laird method based on a random effects model.

## Results

### Search results (see Figure 1 for prisma diagram)

We identified 105 unique publications in the electronic database search, of which 16 met eligibility criteria. One additional paper was included from the references list of included papers, resulting in 17 included papers that reported 16 studies: five took place in the UK, six in other European counties, three in the USA, and one in each of Taiwan, Japan, and Australia. The majority of papers collected information using the Quality of Life-Alzheimer's Disease (QOL-AD) (*n* = 16), with five studies using the Alzheimer's Disease Related Quality of Life (ADRQL); other measures used (each in one paper) were: QoL in Late-Stage Dementia (QUALID); Dementia Quality of Life (DQOL); QUALIDEM; and a single item questionnaire. Five studies measured staff and family carer perspectives; ten studies only asked staff; and one only asked family carers to proxy-rate life quality.

### Methodological quality

There were 16 higher quality papers. One paper did not clearly define how they screened for residents with dementia in the sample (Graske *et al*., 2012) so was rated as lower quality and mentioned in brief at the end. Higher quality study results are presented below.

## Studies comparing staff and family carer proxy QOL scores (*n* = 4)

Four studies (Beer *et al*., 2010; Moyle *et al*., 2012; Crespo and De Quiros, 2013; Clare *et al*., 2014) (total *n* = 1,290) collected data from both staff and relative perspectives. Data are described in Table 1. The total scores for staff and family proxy reports did not differ significantly in our meta-analysis (Figure 2; pooled effect size 0.06 (95% CI = −0.08 to 0.19)) nor in the individual study analyses. One study collected data using two proxy report measures with the same participants (Beer *et al*., 2010); the QOL-AD are included in Figure 2; results using the other measure were very similar. Where correlations between ratings were given (*n* = 3), these are also described in Table 1. In two studies, individual scores were significantly correlated (Beer *et al*., 2010; Clare *et al*., 2014); while Crespo and De Quiros (2013) reported poor agreement between individual ratings (ICC < 0.4).

**Table 1.** Data used in meta-analysis

										correlation
		qol	care	staff	staff	staff	relative	relative	relative	between
paper	location	measure	home *n*	*n*	mean	sd	*n*	mean	sd	ratings
Beer *et al*. (2010)	Australia	QoL-AD	39	324	32.1	7.4	292	32.4	8.2	Not given
Beer *et al*. (2010)	Australia	ADRQL	39	347	72.8	16.3	298	74.9	14.7	*r* = 0.479 *p* < 0.001
Clare *et al*. (2014)	United Kingdom	QUALID	12	105	21.96	6.21	73	21.66	6.71	*r* = 0.412 *p* = 0.000
Crespo and De Quiros (2013)	Spain	QOL-AD	11	197	30.95	7.21	184	29.31	7.57	ICC = 0.298 CI = 0.126–0.468
Moyle *et al*. (2012)	Australia	QOL-AD	4	57	2.34	0.5	58	2.35	0.57	Not given

**Figure 1.**
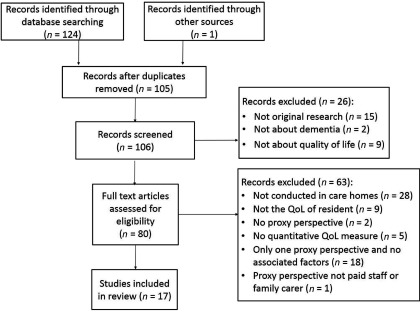
PRISMA 2009 flow diagram.

**Figure 2.**
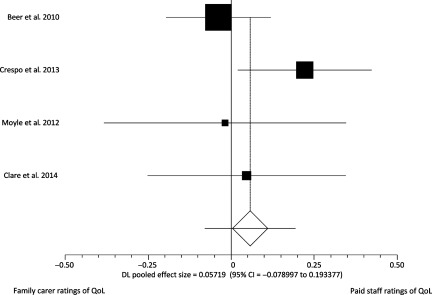
Forest plot.

## Correlates of quality of life

Factors associated with a staff and relative rated QoL are summarized in Table 2 (*n* = 3,537).

**Table 2.** Correlates of quality of life as rated by staff and relatives

					better						
					staff and					better	
		sample	s	r	relative	staff	relative	better	staff	relative	relative
study	measures	description	*n*	*n*	qol	statistic	statistic	staff qol	statistic	qol	statistic
Beer *et al*. (2010)	QoL-AD	*39* Australian RHs	*324*	*292*	+ MMSE − NPI − staff distress − falls	*β* = −3.19 (−5.91, −0.47) *β* = −5.36 (−7.21, −3.52) *β* = −3.76 (−5.71, −1.82) *β* = −5.15 (−7.61, −2.70)	*β* = −4.47 (−7.74, −1.20) *β* = −3.72 (−6.07, −1.37) *β* = −3.99 (−5.39, −0.61) *β* = −4.21 (−7.30, −1.11)	− hospital presentations	*β* = −5.75 (−9.47, −2.03)	− case conference − GP reviews + resident weight + age + pain	*B* = −5.96 (10.27, −1.64) *B* = −3.04 (−5.73, −0.36) *B* = 3.82 (0.03, 7.61) *B* = 2.67 (0.34, 5.01) *B* = 4.58 (1.69, 7.46)
	ADRQL		*347*	*298*	+ MMSE − NPI − staff distress − falls perimeter secure	*β* = −6.90 (−12.06, −1.74) *β* = −16.56 (−19.74, −13.38) *β* = −10.54 (−14.17, −6.91) *β* = −5.56 (−10.59, −0.53) *β* = −7.03 (−11.20, −2.85)	*β* = −5.37 (−10.33, −0.41) *β* = −7.18 (−10.60, −3.76) *β* = −4.64 (−8.18, −1.10) *β* = −4.95 (−9.62, −0.29) *β* = −7.99 (−11.59, −4.39)	+ resident weight + Case conferencing − hospital presentations	*β* = 3.57 (1.10, 6.31) *β* = −5.96 (−10.27,1.64) *β* = −9.26 (−16.41, −2.12)	None	–
Beerens *et al*. (2014)	QoL-AD	*256* LTCs in 8 EU countries	*791*	–	–	–	–	− depressive symptoms − pressure ulcers	*SE* = 0.048, *p* < 0.001 *SE* = 1.232, *p* = 0.01	–	–
Clare *et al*. (2014)	QUALID	*12* LTCs in UK	*105*	*73*	− psychotropic medication	*r* = 0.198, *p* = 0.43	*r* = 0.252, *p* = 0.32	− benzodiazepine medication + greater responsiveness − difficulties in self-care + better behavior	*r* = 0.315, *p* = 0.001 *r* = −0.331, p = 0.005 *r* = 0.256, *p* = 0.008 *r* = 0.362, *p* < 0.001	None	-
Cordner *et al*. (2010)	ADRQL	*3* NHs in USA	*119*	–	–	–	-	Female sex + age − education − behavior problems + identified pain + cognitive function	*t* = −2.36, *p* = 0.02 *r* = 0.28, *p* = 0.002 r *= −0.21, p =0.03* *t* = −2.50, *p* = 0.01 *t* = −3.14, *p* = 0.002 *r* = 0.53, *p* < 0.001	–	–
Crespo *et al*. (2012; Crespo and De Quiros, 2013)	QoL-AD	*11* NHs in Spain	*197*	*184*	+ ADL + cognitive functioning − incontinence − use of nappies − feeding tubes − probes − physical restraint	*r* = 0.511, *p* < 0.001 *r* = 0.258, *p* < 0.004 *r* = −0.450, *p* < 0.001 *r* = -0.313, *p* < 0.001 *r* = −0.285, *p* < 0.001 *r* = −0.187, *p* = 0.009 *r* = −0.306, *p* < 0.001	*r* = 0.378, *p* < 0.001 *r* = 0.255, *p* < 0.009 *r* = −0.304, *p* < 0.001 *r* = −0.200, *p* =0.008 *r* = −0.286, *p* < 0.001 *r* = −0.131, *p* = 0.083 *r* = −0.240, *p* = 0.002	+ staff satisfaction permanent shifts versus rotating shifts private homes versus public homes	*r* = 0.144, *p* = 0.046 *r* = 0.297, *p* < 0.001	+ talks with family −family contribution to NH fees − time resident lived in NH	*r* = 0.298, *p* < 0.001 *r* = −0.330, *p* < 0.001 *r* = −2.09, *p* = 0.005
Graske *et al*. (2014)	ADRQL	*5* NHs in Germany	*88*	–	–	–	–	− dementia severity + nurses satisfaction with life − challenging behaviors − burnout of nurses − days worked prior to ratings	*β* = 11.66 (3.97, 19.36) *β* = 1.37 (0.679, 2.05) *β* = 5.29 (0.33, 10.19) *β* = −10.54 (17.01, −4.06) *β* = 1.28 (0.10, 2.46)	–	–
	QUALIDEM		*88*	–	–	–	–	− challenging behaviors + burden for nurses + satisfaction with life	*β* = 11.48 (5.10, 17.86) *β* = 17.06 (5.71, 28.40) *β* = 1.42 (0.53, 2.31)	–	–
Hoe *et al*. (2006)	QoL-AD	*24* RCS in UK	*224*	–	–			+ ADL − cognitive impairment − challenging behavior − depression − anxiety − unmet needs	*r* = 0.33, *p* < 0.001 *r* = −0.22, *p* < 0.001 *r* = −0.40, *p* < 0.001 *r* = −0.36, *p* < 0.001 *r* = −0.33, *p* < 0.001 *r* = −0.39, *p* < 0.001	–	–
Huang *et al*. (2015)	QoL-AD	*48* NHs in Taiwan	–	*48*	–	–	–	–	–	− depression + high mutuality	*β* = −0.52 (0.83, −0.21) *β* = 0.22 (0.11, 0.33)
Moyle *et al*. (2012)	QoL-AD	*4* LTC in Japan	*58*	*58*	None	–	–	+ ADL	*F* = 7.872, *p* < 0.000	None	–
Nakanishi *et al*. (2011)	QoL-AD	*4* LTCs in Japan	*116*	–	–	–	–	+ ADL + MMSE − dementia severity − NPI No apathy	*r* = 027, *p* < 0.01 *r* = 0.18, *p* < 0.05 *r* = −0.23, *p* < 0.01 *r* = −0.31, *p* < 0.01 *r* = −0.30, *p* < 0.01	–	–
Sloane *et al*. (2005)	QoL-AD	*45* LTCs in USA	*410*	–	–	–	–	+ cognitive function + ADL − agitation − depression	19.8%, *p* < 0.001 21.4%, *p* < 0.001 2.8%, *p* < 0.01 2.0%, *p* < 0.01	–	–
	ADRQL		–	–	–	–	+ cognitive function + ADL − agitation − depression	18.4%, *p* < 0.001 16.4%, *p* < 0.001 10.2%, *p* < 0.001 8.5%, *p* < 0.001	–	–
Spector (2006)	QoL-AD	9 CHs in UK	*76*	–	–	–	–	+ Staff Hope	*R* = −0.072, *p* – 0.03	–	–
Winzelberg *et al*. (2005)	QOL-AD	*38* RHs in USA	*143*	–	–	–	–	+ cognitive function + ADL − behavioral symptoms − work stress + person centered sub scale	*SE* = −5.72, *p* <0.05 SE = −1.18, *p* < 0.001 *SE* = −0.44, *p* < 0.05 *r* = 0.117, *p* < 0.05 *r* = 0.188, *p* < 0.05	–	–
Zimmerman *et al.* (2005)	ADRQL	*45* RHs in UK	*302*	–	–	–	–	− stable resident-staff assignment + observed & reported communication + flexible admission + problem behavior policies + family involvement + contracted staff	Not given, *p* < 0.05 Not given, *p* < 0.05 Not given, *p* < 0.05 Not given, *p* < 0.05 Not given, *p* < 0.001 Not given, *p* < 0.05 Not given, *p* < 0.05 Not given, *p* < 0.05	–	–
	QOL-AD		*301*	–	–	–	–	+ communication − stable resident assignment	Not given, *p* < 0.05 Not given, *p* < 0.05	–	–

## Resident's Physical Health

Lower staff and relative rated QoL was associated with: lower resident weight (Beer *et al*., 2010); the presence of pain (Beer *et al*., 2010; Cordner *et al*., 2010); no use of night time incontinence pads (Crespo and De Quiros, 2013); and falls (Beer *et al*., 2010). Falls were associated with staff, relative, and resident ratings of QoL (Beer *et al*., 2010). Lower staff rated QoL was associated with hospitalization in the last month (Beer *et al*., 2010); there was also a non-significant trend toward an association of hospitalization with lower relative rated QoL. Pressure ulcers were associated with lower QoL for staff, while this association with relative rated QoL was not tested (Beerens *et al*., 2014).

## Resident's mental health

### Staff and relative ratings

Lower staff and relative rated QoL was associated with more neuropsychiatric symptoms, indicated by: the prescription of psychotropic medications (Clare *et al*., 2014); higher neuropsychiatric inventory scores (Beer *et al*., 2010; Nakanishi *et al*., 2011); more anxiety (Hoe *et al*., 2006) and depressive symptoms (Sloane *et al*., 2005; Hoe *et al*., 2006; Nakanishi *et al*., 2011; Crespo and De Quiros, 2013; Winzelberg *et al*., 2005; Huang *et al*., 2015). Symptoms of depression were correlated with staff, resident, and relative rated QoL perspectives (Crespo and De Quiros, 2013).

### Staff ratings

The prescription of benzodiazepine medication showed a small correlation (*r* = 0.315, *p* = 0.001) with staff ratings of QoL but not with relative ratings (*r* = 0.062, *p* = 0.601) (Clare *et al*., 2014). Lower staff rated QoL was correlated with challenging resident behavior (Cordner *et al*., 2010; Clare *et al*., 2014; Graske *et al*., 2014) but not with relative ratings (Clare *et al*., 2014). More agitation was also associated with lower QoL for staff but relative ratings were not measured in this study (Sloane *et al*., 2005).

## Disease progression

### Staff and relative ratings

Both staff and relative ratings of QoL were lower where there was more impairment in Activities of Daily Living (ADL) (Sloane *et al*., 2005; Hoe *et al*., 2006; Nakanishi *et al*., 2011; Crespo and De Quiros, 2013; Winzelberg *et al*., 2005); in one study more impairments in ADL was related to lower staff rated QoL (*F* = 7.872*, p* = 0.001) but this did not reach statistical significance for relative rated QoL (*F* = 2.528, *p* = 0.074) (Moyle *et al*., 2012). Both staff and relative proxy rated QoL lower where there was more impairment in cognition (Sloane *et al*., 2005; Cordner *et al*., 2010; Nakanishi *et al*., 2011; Crespo and De Quiros, 2013; Winzelberg *et al*., 2005). Higher staff rated QoL was correlated with a greater responsiveness to stimuli (*r* = −0.331, *p* = 0.005) but relative ratings were not (*r =* −0.052, *p* = 0.713) (Clare *et al*., 2014).

## Institutional and environmental factors

### Staff and relative ratings

Better communication, indicated by a higher percent of observations in which someone talked to or touched the resident during an observational period, was related to higher staff and relative rated QoL (Zimmerman *et al*., 2005), as was regular staff and family contact (Crespo and De Quiros, 2013) and case conferencing and GP review (Beer *et al*., 2010).

### Staff rated quality of life

Higher staff distress was significantly related to worse QoL as rated by staff and relatives, but the relationship with relative ratings was much smaller (respectively *β* = −10.54 (95% CIs = −14.17, −6.91) and *β* = −4.64 (95% CIs = −8.18, −1.10)) (Beer *et al*., 2010). Staff working permanent shifts rated QoL higher than those working rotating shifts (Crespo and De Quiros, 2013). Additionally, more stable resident: staff assignment was related to lower ratings of QoL (Zimmerman *et al*., 2005) as was a higher number of days worked in advance of the rating QoL of a resident (Graske *et al*., 2014). Furthermore, lower staff rated QoL was associated with: higher staff burnout (Graske *et al*., 2014); high work stress (Winzelberg, 2005); lower nurse satisfaction; (Crespo and De Quiros, 2013; Graske *et al*., 2014); more unmet needs of the residents (Hoe *et al*., 2006); fewer numbers of contract staff (Zimmerman *et al*., 2005); lower scores on the person center subscale (Winzelberg *et al*., 2005); less acceptance of problem behavior policies (Zimmerman *et al*., 2005); and the type of center administration, where residents in public homes were rated as having lower QoL than those in private homes (Crespo and De Quiros, 2013).

### Relative rated quality of life

Relative rated QoL was negatively associated with more documented (*β* = −3.38 (CIs −6.66, −0.10)) and observed restraint (*β* = −6.21 (CIs −10.80, −1.62)) but the relationship with staff ratings was much smaller and did not reach significance (*β* = −1.65 (CIs −6.94, 3.65)) (Beer *et al*., 2010). Better relative rated QoL was associated with the family not making a financial contribution to nursing home fees (Crespo and De Quiros, 2013), and the resident having spent less time living in the nursing home (Crespo and De Quiros, 2013) but analysis comparing these to staff ratings was not conducted.

### Lower quality paper

Graske *et al*. (2012) reported mean differences and found that only for the domains “physical health” and “life as a whole” the staff rated QoL higher than the self rated QoL and that staff rated the following domains lower than residents: “memory” (0.51, p < 0.05), “family” (0.36, *p* < 0.05), “marriage” (0.49, *p* < 0.05), “friends” (0.75, *p* < 0.05), “ability to do chores” (0.34, *p* < 0.05), and “ability to do things for fun” (0.32, *p* < 0.05). This paper also found that if the primary nurse rated the QoL, there was significantly more agreement with resident ratings (*p* < 0.05).

### Factors associated with change in quality of life

Beerens *et al*. (2015) found that better cognitive abilities at baseline were associated with a decrease in self-reported QoL (*SE* = 0.049, *p* < 0.05) over a three month period. In contrast, greater dependency (*SE* = 0.320; 95% CIs 1.082, 0.194) and more depressive symptoms (*SE* = −0.042; 95% CIs −0.118, 0.083) at baseline were associated with declining staff proxy-reported QoL.

## Discussion

When comparing the overall means of groups we did not find a significant difference between global QoL scores between relative and staff proxy ratings for care home residents with dementia. In three of four studies examining this, there was a non-significant trend toward care staff rating QoL higher than family members, but we can conclude from existing data that any systematic difference in global ratings is small, and not of the magnitude of those reported between self and proxy reports of life quality in people with dementia. The majority of studies included used the QOL-AD and ratings from different proxy groups have not been compared for a number of other QoL measures used in people with dementia. This is an important consideration when evaluating validity of QoL measures for use in care homes. When papers compared ratings for individuals by looking at the correlation between staff and family carer rated QoL there was moderate agreement in two of three papers. These results suggests raters are considering similar things when rating QoL but that there are also some differences that are not reflected in the overall mean score of groups.

Relatives and staff proxy QoL ratings share a clear relationship to resident physical and mental health, including: lower weight, use of antipsychotic medication, depression, higher physical disability, pain, poorer cognitive function, and lower capacity to carry out ADL. Rater-specific factors were also associated with the scores they give. Staff QoL ratings were associated with their own levels of stress and burnout. This fits with existing research in the community that shows that low proxy rated QoL is strongly influenced by the family carer's mood and experience of caring (Karlawish *et al*., 2001; Logsdon *et al*., 2002; Thorgrimsen *et al*., 2003; Sands *et al*., 2004). It is unsurprising that staff who experience the care home where they work as stressful and overwhelming evaluate the life quality of its residents lower. Understanding the impact of rater well-being is a potentially important consideration when evaluating the validity of proxy rated QoL.

The fact that staff rated the QoL of residents who exhibited more agitation, challenging behavior and unmet needs lower, but relatives did not, could suggest that there are aspects of the resident's life that relatives are less aware of, or that staff's own feelings affect their ratings. Agitated and challenging behavior may be more likely to occur at times of personal care or may be more common when relatives are not there, thus, limiting a relatives exposure to these behaviors. Care home studies more commonly measure staff rated QoL, so associations with relative rated QoL have been less widely studied. The relevance of the context of the proxy rater was also demonstrated by the finding that lower relative proxy ratings of QoL were associated with a longer stay in the care home, as well as higher relative contribution to nursing home fees; possibly indicating burden for the relative.

These results suggest it is important to collect data about the environment of the residents as it may explain some of the variation in ratings, particularly in samples with participants recruited from different care homes. Many care home residents with dementia do not have a family member who visits regularly; findings from studies of those that do in which proxy-ratings can be compared, can help us interpret and validate staff rated QoL measurements that are potentially available for all residents.

## Conclusion

Existing research suggests there is little difference between paid carer and family carer perspectives of QoL when comparing overall means scores of the QOL-AD and QUALID but that this different does not imply ratings are the same as they are not strongly correlated. This can be explained by the fact that different factors are associated with proxy reported QoL for staff and family members. Paid carer and family carer proxy rated QoL is lower with the presence of more stress in their own life and this may lead to differences in overall ratings in other more detailed QoL questionnaires or in differences between individuals and this should be explored in future research.

Proxy rated QoL is a vital outcome in a care home context and it is important to understand what is being measured. Future research should investigate the differences between different perspectives of QoL using different QoL measures that allow cost calculations. Cost effectiveness is an important outcome to ensure that interventions give value for money and the DEMQOL (Smith *et al*., 2007) allows this calculation. Further research should investigate whether there is a difference between paid staff and relative proxy rated QoL using the DEMQOL in care homes and the factors associated with all perspectives on QoL. Further increasing our understanding of the concept of QoL will enable us to explore how to maximize QoL for people living with dementia.

## Conflict of interest

None.

## Description of the authors’ roles

Sarah Robertson conducted the literature searches, data extraction, study quality appraisal, and prepared the first draft of the paper as part of her PhD project. Aisling Stringer and Olivia Hamilton independently conducted study quality appraisal and resolved any disagreements with Sarah Robertson. Gill Livingston, Claudia Cooper, and Juanita Hoe revised the paper critically and approved the final version for publication.
